# Neoadjuvant immunochemotherapy improves clinical outcomes of patients with esophageal cancer by mediating anti-tumor immunity of CD8+ T (Tc1) and CD16+ NK cells

**DOI:** 10.3389/fimmu.2024.1412693

**Published:** 2024-07-15

**Authors:** Yunlong He, Depeng Yang, Xiaoyu Lin, Jinfeng Zhang, Rui Cheng, Liangyu Cao, Lijun Yang, Mengmeng Zhang, Xinyue Shi, Xiyun Jin, Handi Sun, Haoxiu Sun, Jingyu Zang, Yu Li, Jianqun Ma, Huan Nie

**Affiliations:** ^1^ School of Life Science and Technology, Harbin Institute of Technology, Harbin, Heilongjiang, China; ^2^ Department of Radiation Oncology, Harbin Medical University Cancer Hospital, Harbin, Heilongjiang, China; ^3^ Department of Thoracic Surgery, Esophagus and Mediastinum, Harbin Medical University Cancer Hospital, Harbin, Heilongjiang, China; ^4^ State Key Laboratory for Conservation and Utilization of Bio-Resource and School of Life Sciences, Yunnan University, Kunming, Yunnan, China; ^5^ School of Interdisciplinary Medicine and Engineering, Harbin Medical University, Harbin, Heilongjiang, China

**Keywords:** esophageal cancer, neoadjuvant immunochemotherapy, tumor microenvironment, CD8+ T cells, CD16+ NK cells, CD276

## Abstract

**Background:**

Esophageal cancer (ESCA) is one of the most common tumors in the world, and treatment using neoadjuvant therapy (NT) based on radiotherapy and/or chemotherapy has still unsatisfactory results. Neoadjuvant immunochemotherapy (NICT) has also become an effective treatment strategy nowadays. However, its impact on the tumor microenvironment (TME) and regulatory mechanisms on T cells and NK cells needs to be further elucidated.

**Methods:**

A total of 279 cases of ESCA who underwent surgery alone [non-neoadjuvant therapy (NONE)], neoadjuvant chemotherapy (NCT), and NICT were collected, and their therapeutic effect and survival period were compared. Further, RNA sequencing combined with biological information was used to analyze the expression of immune-related genes. Immunohistochemistry, immunofluorescence, and quantitative real-time PCR (qRT-PCR) were used to verify the activation and infiltration status of CD8+ T and CD16+ NK cells, as well as the function and regulatory pathway of killing tumor cells.

**Results:**

Patients with ESCA in the NICT group showed better clinical response, median survival, and 2-year survival rates (*p* < 0.05) compared with the NCT group. Our RNA sequencing data revealed that NICT could promote the expression of immune-related genes. The infiltration and activation of immune cells centered with CD8+ T cells were significantly enhanced. CD8+ T cells activated by PD-1 inhibitors secreted more IFN-γ and cytotoxic effector factor cells through the transcription factor of EOMES and TBX21. At the same time, activated CD8+ T cells mediated the CD16+ NK cell activation and secreted more IFN-γ to kill ESCA cells. In addition, the immunofluorescence co-staining results showed that more CD276+ tumor cells and CD16+ NK cells were existed in pre-NCT and pre-NICT group. However, CD276+ tumor cells were reduced significantly in the post-NICT group, while they still appeared in the post-NCT group, which means that CD16+ NK cells can recognize and kill CD276+ tumor cells after immune checkpoint blocker (ICB) treatment.

**Conclusion:**

NICT can improve the therapeutic effect and survival period of resectable ESCA patients. NICT could promote the expression of immune-related genes and activate CD8+ T and CD16+ NK cells to secrete more IFN-γ to kill ESCA cells. It provides a theoretical basis and clinical evidence for its potential as an NT strategy in ESCA.

## Introduction

Esophageal cancer (ESCA) is still one of the leading causes of cancer-related deaths worldwide. If the incidence rate of ESCA remains stable, 957,000 new cases and 880,000 deaths will occur globally by 2040. Effective treatment is crucial in reducing ESCA-attributable mortality (1). Neoadjuvant therapy (NT) has become a first-line treatment option for many types of cancer in recent years, and its application in ESCA is also widespread (2, 3). However, commonly used neoadjuvant chemotherapy (NCT) and neoadjuvant radiochemotherapy (NRCT) still cannot meet people’s expectations for therapeutic effects. Therefore, more optimized treatment strategies are still needed to address the current treatment challenges of esophageal cancer.

Immune checkpoint blockers (ICBs) have transformed the landscape of cancer therapy, providing substantial benefits to patients. Remarkable progress has been achieved in the field, particularly in the treatment of non-small cell lung cancer (NSCLC), gastric cancer, and liver cancer, among others (4–6). At present, the combination of ICBs and neoadjuvant chemotherapy, i.e., neoadjuvant immunochemotherapy (NICT), has been explored in therapy for ESCA. Several clinical trials have been initiated and improved survival in esophageal squamous cell carcinoma (ESCC) patients who received NICT (7, 8). Although some clinical evidence currently demonstrates the advantages of NICT, its specific mechanism is still unclear and has been a research hotspot (9). NICT can activate T cells in cancer patients and significantly improve disease remission rates by blocking signals that inhibit T-cell activation, which is one of the primary mechanisms of current clinical tumor immunosuppressive therapies (10). Some research has found that NICT could enhance anti-tumor immune responses by activating CD4+ and CD8+ T lymphocytes (11, 12). Activated CD8+ T cells subsequently kill tumor cells by mediating the release of lymphotoxin (LT) from a subset of cytotoxic T cells (Tc1) (13). Additionally, ICBs have the capability to activate NK cells, promoting the recognition of tumor cell surface antigens and the destruction of antibody-bound tumor cells (14). Moreover, activated NK cells could mediate apoptosis of tumor cells through the expression of transcription factors TBX21 and EOMES, factors that can regulate the high expression of cytokines (e.g., IFN-γ and TNF-α) and exert anti-tumor effects through immunomodulatory effects (15). Until now, NK cells have been used as feasible immunotherapy in several clinical trials (16–18). In the context of anti-tumor immunotherapy, ICBs enhance NK cell activation and cytotoxicity by increasing the frequency of CD16+ NK cells (19). Therefore, T cells and NK cells play complementary roles in tumor immunity, and their combination provides opportunities to deepen the impact of immunotherapy. However, further research is still required to elucidate the roles and effect details of CD8+ T and CD16+ NK cells in NICT.

Programmed cell death protein 1 (PDCD1, PD-1, CD279) and programmed cell death ligand 1 (PD-L1, CD274/B7-H1) are regarded as key targets for anti-tumor immunotherapy currently (20, 21). B7-H1 belongs to one of the members of the B7 immunoglobulin superfamily, which is closely related to tumor progression and plays a crucial role in tumor immunity (22, 23). However, given the fact that the response to treatment with ICBs does not always correlate with PD-L1 expression, some ESCA patients may not benefit from immunotherapy (24, 25). Therefore, it would be a meaningful subject in immunotherapy to find novel B7 immunoglobulin superfamily members serving as targets for ICBs. The B7 homolog 3 protein (B7-H3, CD276) of the B7 immunoglobulin superfamily, like its cognate member PD-L1, plays a crucial role in regulating tumor immune responses (26). CD276 has been found to be overexpressed in numerous solid tumors with very minimal expression in their corresponding normal tissues. In most tumors, high expression of CD276 is strongly associated with cancer progression and poor prognosis for cancer patients. Therefore, CD276 has been considered a promising target for immunotherapy research studies (27). However, the receptor for CD276 has remained unidentified, and the role of CD276 in immunotherapy remains controversial (28).

Herein, 279 cases of surgical ESCA patients were reviewed, and their efficacy and survival after undergoing NCT and NICT were compared. Further, the expressions of immune-related genes and the changes in the tumor immune microenvironment (TIME) were analyzed pre- and post-NT. The functions and regulatory pathways of CD8+ T and CD16+ NK cells were elucidated in EC tissues of the NICT group. Therefore, our research may lay a foundation for the mechanism research and serve as strong evidence of NICT in ESCA.

## Materials and methods

### Patients and samples

Clinical data were collected from 279 patients who underwent radical ESCA surgery from January 2017 to December 2022 in the same hospital. This study was approved by the ethics committee of the hospital (2020–50-IIT), and all the patients signed the informed consent form. The sample size was 120 patients for the NONE group, 64 patients for the NCT group, and 95 patients for the NICT group. The basic characteristics of the patients are listed in **Supplementary Table S1**, with no major differences across groups including sex, age, and smoking behavior. The patients from the NCT and NICT groups underwent one to four cycles of NT [i.e., paclitaxel for injection (albumin bound), platinum as chemotherapy drugs, and PD-1 inhibitors as ICBs] before surgery. A number of cancer and para-cancer tissue samples were collected from the included cases and stored in liquid nitrogen.

In accordance with the National Comprehensive Cancer Network (NCCN) guidelines and the patient’s status, the follow-up appointments were scheduled, with the exception of those of four missing patients in the NCT group.

### Acquisition of imaging data

With the assistance of imaging experts, imaging analyses, including CT, MRI, and PET/CT, were performed pre- and post-NT as well as pre- and post-surgery among all included cases. Attention was focused on the longitudinal diameter and the maximum pipe-wall thickness of the tumors, and the product of these two values was used as an auxiliary criterion for determining the changes in the tumor size pre- and post-NT. The imaging data came from the hospital’s database.

### RNA isolation, RNA sequencing, and data analyses

The clinical tissues of ESCA (50 mg) were sectioned using a cryostat microtome, and total RNA was exacted with TRIzol reagent (#15596018, Invitrogen, Carlsbad, CA, USA). After passing the quality control, the total RNA samples were sequenced, and the data analyses were performed. The differentially expressed genes were shown with fold change |log(FC)| > 1 and *p* < 0.01. All statistical analyses were conducted using the R software (Version 4.0.2). Kyoto Encyclopedia of Genes and Genomes (KEGG) enrichment and Gene Ontology (GO) function annotation analyses were performed using the R package “clusterProfiler”. KEGG or GO terms with BH-corrected *p* < 0.05 were considered significant. “Enrichplot” was used to visualize the significant results. Gene Set Enrichment Analysis (GSEA) function in R was applied to analyze the gene expression condition and identify the enrichment status of the gene set (i.e., NICT/NCT, NICT/NONE, and NCT/NONE). Specifically, the gene set of |NES| > 1 and false discovery rate (FDR) q < 0.25 were identified with the most remarkable changes.

### The scoring of immune cell infiltration

Employing the “estimate” package of R, we scored the gene expression matrix of stromal and immune cells from different tumor tissues, as well as for the immune infiltration. The scoring of immune infiltration is positively associated with the proportion of immune cell infiltration within the tumor tissues.

### The scoring of cell cytotoxicity

The scoring of cell cytotoxicity was applied to analyze the cytotoxicity of infiltrating immune effector cells including CD8+ T and CD16+ NK cells. The geometric mean of the expression quantity of GZMM and PRF1 was adopted to reflect the scoring of cell cytotoxicity (CYT) for the tumor tissue of included patients.

### The correlation between T-cell surface marker and activity marker

The correlation between T-cell surface marker and activity marker was analyzed utilizing ESCA samples from the Gene Expression Profiling Interactive Analysis (GEPIA) database (http://gepia.cancer-pku.cn/index.html), and Pearson’s correlation coefficient was employed to assess the correlation between T-cell surface marker (i.e., CD8A and CD8B) and activity marker (i.e., EOMES, TCIRG1, GZMA, GZMM, PRF1, and IFN-γ).

### Quantitative real-time PCR

Total RNA from ESCA tissues was reverse-transcribed into cDNA using the PrimeScript RT-PCR Kit (#RR047A, Takara Biotechnology, Mountain View, CA, USA). Quantitative real-time PCR (qRT-PCR) was conducted using the FastStart Universal SYBR Green Master (#04913914001, Roche, Basel, Switzerland) on QuantStudio 3 (Applied Biosystems, Foster City, CA, USA). Primers for qRT-PCR are described in **Supplementary Table S2**. The mRNA level of β-actin was used as an internal control.

### H&E staining and immunohistochemical staining

The paraffin sections (5 μm) of ESCA tissues were de-paraffinized, dehydrated by xylene and alcohol, and stained with hematoxylin and eosin (H&E) for each case. Other sections were used for immunohistochemical staining with specific antibodies. After antigen retrieval was performed with 10 mM Tris-EDTA (pH 9.0) in microwave for 10 minutes, the tissue slides were treated with 3% hydrogen peroxide for 15 minutes and blocked with 3% bovine serum albumin (BSA) for 1 hour at room temperature (RT). The slides were incubated with primary antibody at 4°C overnight. The CD8 (66868–1-lg), TCIRG1 (12649–1-AP), CD16 (66779–1-lg), and CD276 (14453–1-AP) antibodies were purchased from ProteinTech (Chicago, IL, USA) (**Supplementary Table S3**). Next, slides were incubated with a secondary antibody for 1 hour at RT and exposed to a diaminobenzidine (DAB) staining solution. Afterward, nuclei were stained with hematoxylin. Finally, the slides were mounted with neutral balsam and observed under a zoom stereo microscope (Axio Zoom.V16, Zeiss, Oberkochen, Germany).

### Immunofluorescence staining

The ESCA tissue slides initially were blocked with 3% BSA for 1 hour at RT followed by overnight incubation with the primary antibody at 4°C. The IFN-γ (15365–1-AP), CD8 (66868–1-lg), TCIRG1 (12649–1-AP), CD16 (66779–1-lg and 16559–1-AP), and CD276 (14453–1-AP and 66481–1-lg) antibodies were purchased from ProteinTech (**Supplementary Table S3**). Then, they were incubated with a secondary antibody for 1 hour at RT after the slides were de-paraffinized and dehydrated with xylene and alcohol. The signals from specific antibodies were labeled with Fluorescein Isothiocyanate (green) and Rhodamine (red) and then re-stained with 4′,6-diamidino-2-phenylindole (DAPI) for 10 minutes at RT, and slides were mounted with 90% glycerin and kept in the dark. The slides were visualized under a confocal microscope (OLYMPUS, Tokyo, Japan; FV3000) and a fluorescence microscope (ECHO RVL2-K2).

### Data source

Expression data for genes including PRF1, GZMA, and GZMM across various immune cells were obtained from “The Human Protein Atlas data analysis” (HPA: https://www.proteinatlas.org/). ESCA RNA sequencing (RNA-seq) data were obtained from The Cancer Genome Atlas (TCGA) database (https://cancergenome.nih.gov/) and ESCC data (GSE145370) from the Gene Expression Omnibus (GEO) database (https://www.ncbi.nlm.nih.gov/geo/). The list of genes related to signaling pathways was obtained from the MSigDB database (https://www.gsea-msigdb.org/gsea/msigdb/index.jsp).

### Statistical analysis

In this study, data for continuous variables are presented as the mean ± standard of measurement in at least three independent experiments, and median [95% confidence interval (CI)] was used when sample distribution was non-normal. Statistical significance was determined using a two-tailed Student’s t-test. For comparison of categorical variables between groups, chi-square tests in cross-tabulation were used. Overall survival (OS) was compared using the Kaplan–Meier curve analysis and log-rank tests. Median percentage reduction with a 95% CI was applied to reflect the reduction in the size of the tumor for each treatment group before and after NT. Statistical analysis was performed using IBM SPSS Statistics 27, and *p*-value <0.05 was considered statistically significant (*, *p* < 0.05; **, *p* < 0.01; ***, *p* < 0.001). For figures, statistical tests were justified as appropriate. Bars in the graphs represent mean ± SEMs. Analyses and graphical presentations were performed using the GraphPad Prism 9.5 software.

## Results

### NICT significantly reduces tumor stage and lesion size

**Figure 1:**
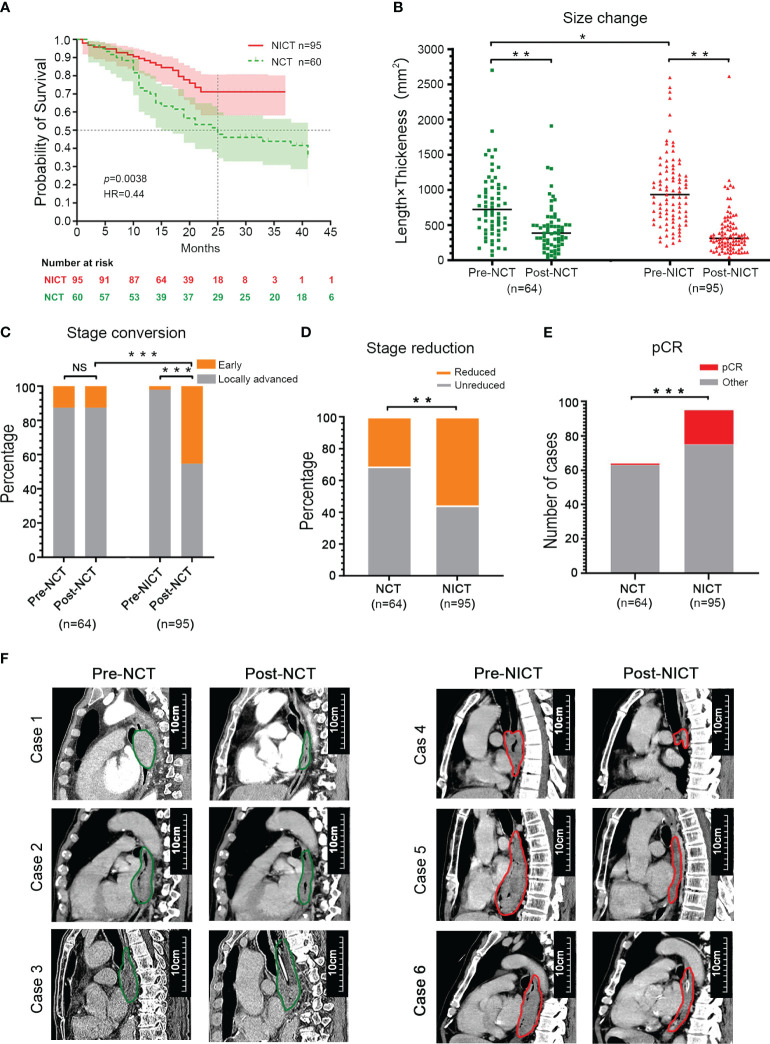
NICT significantly improved the therapeutic effect of esophageal cancer (ESCA). **(A)** Survival period. NICT, neoadjuvant immunochemotherapy; NCT, neoadjuvant chemotherapy; HR, hazard ratio. **(B)** Changes in tumor size. Pre-NCT, pre-neoadjuvant chemotherapy; Post-NCT, post-neoadjuvant chemotherapy; Pre-NICT, pre-neoadjuvant immunochemotherapy; Post-NICT, post-neoadjuvant immunochemotherapy. **(C)** Conversion rate of clinical stage. **(D)** The reduction rate of clinical stage. **(E)** pCR, pathologic complete response. **(F)** Clinical cases. The maximum longitudinal diameters of tumors in the sagittal plane of CT images. NCT group (left): Case 1 and Case 2 had partial response (PR), and Case 3 had progressive disease (PD). NICT group (right): Case 4 had pCR, and Case 5 and Case 6 had PR. *p < 0.05, **p < 0.01, ***p < 0.001. NS, no significance.

As an important type of ICB, PD-1 inhibitors have been widely applied in clinical tumor treatment and NT for ESCA due to their effectiveness. To assess the clinical treatment effect of NT with PD-1 inhibitors, as well as to elucidate the immune activity and recognized target of PD-1 inhibitors in ESCA treatment, our study first compared the survival rates of patients who received NT using follow-up and survival analysis. As depicted in **Figure 1A**, the survival rates of the NICT (n = 95) and NCT groups (n = 64) were 70.9% and 51.5% (within 2 years), respectively. The NCT group exhibited a median survival period of 25 months, while no median survival period was determined for the NICT group due to its higher survival rates, which showed statistical significance [*p* < 0.005, hazard ratio (HR) = 0.44]. The survival analysis indicated that the treatment effect of the NICT group is superior to that of the NCT group. Then, the changes in tumor size pre- and post-NT were analyzed using the longitudinal diameters (mm) × maximum thickness (mm) of the tumor using the data of CT images. The NONE group had the minimum size of tumor (514.08 ± 439.86 mm^2^), followed by the NCT group (819.17 ± 566.65 mm^2^) and the NICT group (1,063.62 ± 683.50 mm^2^) before treatment. The median size of the NICT group was significantly larger than that of the NONE group and the NCT group (*p* < 0.05) (**Supplementary Figure S1A**, **Table 1**). After NT, higher-level reduction was found in the NICT group (reduced 63.42%) as compared with the NCT group (reduced 43.52%), although the mean size of the tumor in both the NCT and NICT groups significantly reduced (**Figure 1B**, **Supplementary Figure S1B**). That is to say, the NICT group with larger tumors before treatment had smaller tumors after treatment. Subsequently, the differences in clinical staging among different groups were compared. Before NT, there were 56 (87.5%) and 93 (97.9%) cases classified as later staging in the NCT and NICT groups, respectively, along with eight (12.5%) and two (2.1%) cases classified as earlier staging (*p* < 0.001). After NT, apart from three cases who did not receive endoscopic examination or were without clear definitions of clinical staging in the NCT group, for the NCT and NICT groups, there were respectively 53 (82.8%) and 52 (54.7%) later clinical staging cases, along with eight (12.5%) and 43 (45.3%) earlier clinical staging cases (*p* < 0.001); see **Table 2** and **Figure 1C**. Thus, in the NICT group, more cases were converted to an early stage, with a significant reduction in the number of locally advanced cases, while this conversion was not significant in the NCT group. Meanwhile, compared with the NCT group, more cases in the NICT group showed a reduction of clinical stage post-NT (n = 53 55.8% vs. n = 20, 31.3%), which was significantly statistically different (**Table 2** and **Figure 1D**, *p* = 0.005). Additionally, 10 (10.5%) cases showed clinical stage advancement in the NICT group, while there were 20 (31.3%) cases in the NCT group (**Table 2** and **Supplementary Figure S1C**, *p* < 0.001). Furthermore, there were more cases of pathologic complete response (pCR) in NICT (NICT vs. NCT, n = 1 1.6% vs. n = 21 22.1%), as shown in **Figure 1E**.

**Table 1:** Comparison of changes in tumor size between groups.

Parameters	NONE (%) (n = 120)	NCT (%) (n = 64)	NICT (%) (n = 95)	*p*-Value
Pre-treatment				
Length^a^ (mm)	40.47 ± 17.50	48.86 ± 18.68	60.89 ± 20.53	
Thickness^b^ (mm)	12.10 ± 5.81	15.59 ± 6.06	16.78 ± 6.83	
Length × Thickness (mm^2^)	514.08 ± 439.86	819.17 ± 566.65	1,063.62 ± 683.50	NONE vs. NICT <0.01 NCT vs. NICT 0.019
Post-NT				
Length (mm)		37.03 ± 14.92	39.22 ± 17.81	
Thickness (mm)		11.18 ± 5.16	9.74 ± 5.67	
Length × Thickness (mm^2^)		449.05 ± 336.14	420.05 ± 438.05	0.655
Reduction cases No. (%)				
Length		48 (75%)	84 (88.4%)	0.027
Thickness		56 (87.5%)	92 (96.8%)	0.023
Length × Thickness		59 (92.2%)	95 (100%)	0.006

NONE, non-neoadjuvant therapy; NCT, neoadjuvant chemotherapy; NICT, neoadjuvant immunochemotherapy; NT, neoadjuvant therapy.

a Length, the maximum longitudinal diameter.

b Thickness, the maximum pipe-wall thickness.

**Table 2:** Comparison of changes in clinical stage between NCT and NICT groups.

Parameters	NCT (%) (n = 64)	NICT (%) (n = 95)	*p*-Value
Pre-NT^a^			
I	8 (12.5)	2 (2.1)	0.008
II	18 (28.1)	28 (29.5)	
III	26 (40.6)	58 (61.1)	
IV	12 (18.8)	7 (7.4)	
Could not be determined	0 (0)	0 (0)	
Post-NT^b^			
I	8 (12.5)	43 (45.3)	<0.001
II	17 (26.6)	13 (13.7)	
III	26 (40.6)	38 (40.0)	
IV	10 (15.6)	1 (1.1)	
Could not be determined	3 (4.7)	0 (0)	
Stage reduction Stage progression	20 (31.3) 20 (31.3)	53 (55.8) 10 (10.5)	0.005 <0.001
pCR^c^	1 (1.6)	21 (22.1)	<0.001

NCT, neoadjuvant chemotherapy; NICT, neoadjuvant immunochemotherapy.

a Pre-NT, pre-neoadjuvant therapy.

b Post-NT, post-neoadjuvant therapy.

c pCR, pathologic complete response.

The cross-sectional and longitudinal images of CT provided evidence of the changes in tumor size pre- and post-NT among the NCT and NICT groups, suggesting that the tumor size of the NICT group was mostly larger pre-NT, while it reduced dramatically post-NT, compared with the NCT group (**Figure 1F**, **Supplementary Figure S1D**).

These clinical findings indicated that the NICT group had a better therapeutic effect than the NCT group based on differences in survival period, tumor response, and changes in clinical stage.

### NICT promoted the expression of immune-related genes in esophageal cancer tissues

**Figure 2:**
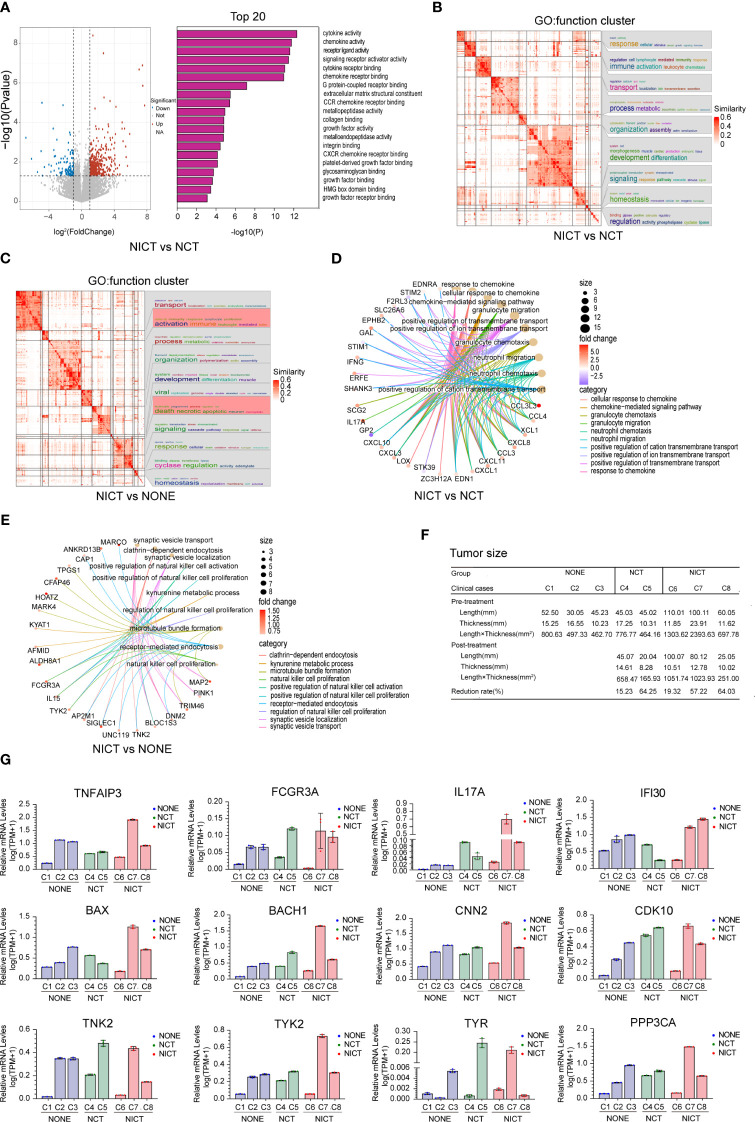
NICT promoted the expression of immune-related genes in ESCA tissues. **(A)** Pathway enrichment analysis of the cytokine, chemokine, and their receptors in NICT group compared to NCT group using RNA sequencing technique. **(B)** The function cluster analysis of Gene Ontology (GO) showing comparison of the differential genes between the NCT and NICT groups. **(C)** The function cluster analysis of GO showing comparison of function clusters of the differential genes between the NONE and NICT groups. **(D)** Comparison of cycle net plot between the NCT and NICT groups. **(E)** Comparison of cycle net plot between the NONE and NICT groups. **(F)** The table presents the tumor sizes pre- and post-treatment for eight clinical cases (from C1 to C8) from the NONE, NCT, and NICT groups. **(G)** qRT-PCR results of 12 immune-related genes and immune-regulated genes in clinical cases among the NONE, NCT, and NICT groups. NICT, neoadjuvant immunochemotherapy; ESCA, esophageal cancer; NCT, neoadjuvant chemotherapy; NONE, non-neoadjuvant therapy; qRT-PCR, quantitative real-time PCR.

To evaluate the characteristics of the immune response of the ESCA tissues after NICT and NCT, we applied the RNA sequencing technique to compare the differences in gene expression of ESCA tissues after NT, where the NONE group was treated as the control group. GO analysis revealed that the cytokine, chemokine, and their receptors in the ESCA tissues from the NICT group (as compared to the NCT group) were more active (**Figure 2A**). The NICT group also exhibited positive activity of the NK cells as compared to the NONE group (**Supplementary Figure S2A**). On the contrary, as compared to the NONE group, the enriched signal pathways in the NCT group involved non-coding RNA (ncRNA) processing, DNA, and mRNA metabolic process (**Supplementary Figure S2B**). Meanwhile, the function cluster of the differential genes from the groups suggested that lymphocyte-mediated immune response and activation immune of lymphocyte-mediated killer immunity in the NICT group were significantly enhanced (**Figure 2B**), compared to the NONE and NCT groups (**Figure 2C**, **Supplementary Figure S2C**). The results of the cycle net plot from the NICT group elucidated the signaling pathway and differential genes associated with positive regulation of NK cell activation and proliferation, and cellular response to chemokines (**Figures 2D, E**, **Supplementary Figure S2D**). Based on the differential genes, we constructed the network of regulating immune function. The network results further suggested that the increased expressions of genes in the NICT group promoted adaptive immune response based on somatic recombination of immune receptors built from the immunoglobulin superfamily domain, activation of immune response, lymphocyte and leukocyte proliferation, and positive regulation of immune cell–cell adhesion (**Supplementary Figure S2E**), as well as activation and positive regulation of NK cells (**Supplementary Figure S2F**).

To validate the relationship between these changes and the immunotherapy for ESCA, we first analyzed the therapeutic effects of each sample (i.e., the response of an individual to treatment, estimating the reduction rate of the length × thickness of the tumor, **Figure 2F**). Then, we compared the expression of immune-related genes in different post-treatment samples. The results of the immune-related gene qRT-PCR are shown in **Figure 2G**. In the NONE group, the detected genes of the case with a larger size of the tumor (i.e., Case 1) showed a low-level expression. In the NICT group, these detected genes were highly expressed in Case 7 with better therapeutic effect (reduced size by 57.22%), while they showed a low-level expression in Case 6 with insignificant size reduction (by 19.32%) after NT.

Based on the findings demonstrated above, we analyzed the GSE145370 dataset from the GEO database according to the markers of T cells and NK cells (**Supplementary Figure S2G**). The analysis results suggested that the predominant immune-infiltrating cells in esophageal cancer tissue are T cells. Compared to the normal tissues, the presence of NK cells reduced dramatically (*p* < 0.01, **Supplementary Figures S2H, I**).

It can be seen that NICT could promote the expression of immune-related genes and activate T cells and NK cells in ESCA tissues.

### NICT activated CD8+ T and NK cells of esophageal cancer tissues

**Figure 3:**
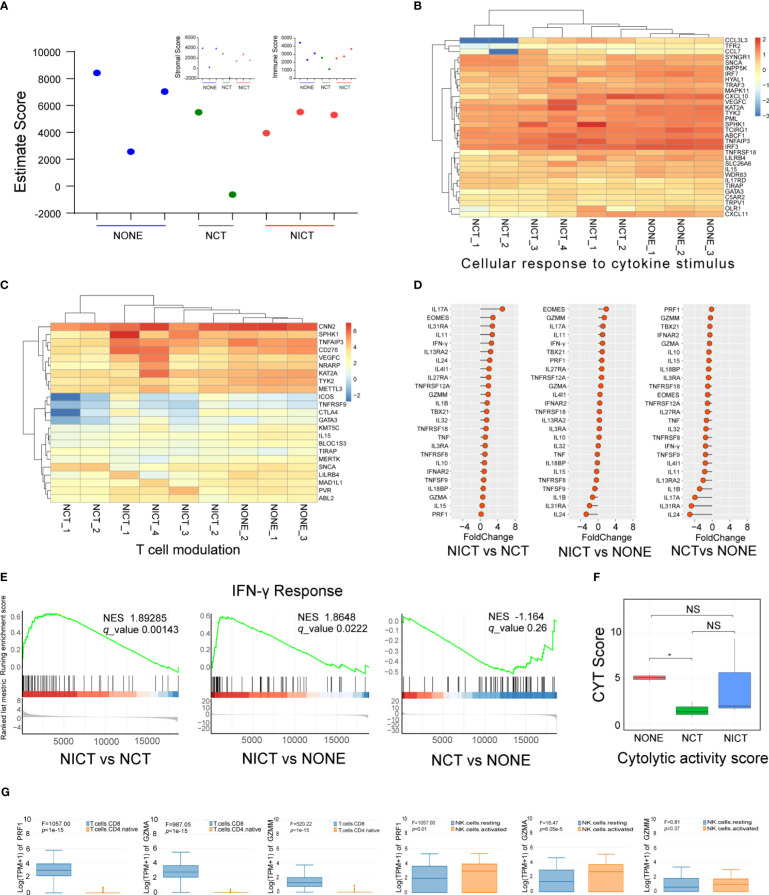
NICT activated the immune microenvironment of ESCA tissues. **(A)** Using ESTIMATE Score to analyze the scoring of immune cell infiltration among NONE, NCT, and NICT groups. **(B)** Heatmap showing the production of cellular response to cytokine stimulus in each group of clinical cases. **(C)** Heatmap showing the production of T-cell modulation in each group of clinical cases. **(D)** Comparison of the expression of cytokines in the NONE, NCT, and NICT groups, especially the cytotoxic effector factors specifically expressed in T cells and NK cells. **(E)** Comparison of IFN-γ response pathways among NONE, NCT, and NICT groups by Gene Set Enrichment Analysis (GSEA). **(F)** Evaluation of the cytotoxic activity of immune cells in tumor tissues by calculating cytolytic activity score (CYT) using PRF1 and GZMM. **(G)** Using HAP database to identify the types of T cells and NK cells expressing PRF1, GZMA, and GZMM in ESCA. NICT, neoadjuvant immunochemotherapy; ESCA, esophageal cancer; NONE, non-neoadjuvant therapy; NCT, neoadjuvant chemotherapy. *p < 0.05, NS, no significance.

In order to assess the regulating effect of NICT on the tumor microenvironment (TME), we applied RNA sequencing data to analyze the status of immune cell infiltration in ESCA tissues. We employed the ESTIMATE Score to acquire the scoring of immune cell infiltration and compared the number of immune cells and differences in activity among the NONE, NCT, and NICT groups (**Figure 3A**). The results showed the NICT group revealed a relatively stable high level of immunity. As compared to the NCT group, the function cluster of GO analysis showed that significant changes were observed in the cellular response to cytokine stimulus (**Figure 3B**), T-cell modulation (**Figure 3C**), protein phosphorylation, and the expression of kinases (**Supplementary Figure S3A**, |log(FC)| > 1, *p* < 0.05). More important, compared with the NCT and NONE groups, differences in the expression of chemokines, interleukins, and killing effector factors had caught our attention in the NICT group (**Figure 3D**, **Supplementary Figure S3B**, fold change > 1, *p* < 0.05).

PRF1, GZMA, and GZMM (i.e., cytotoxic effector factors specifically expressed in T cells and NK cells; **Supplementary Figures S3C, D**) were significantly suppressed in the NCT group, while they increased in the NICT group (**Figure 3D**). Moreover, we evaluated the cytotoxic activity of immune cells in tumor tissues by the cytolytic activity score (CYT) or the marker molecules using PRF1 and GZMM (**Figure 3F**). This finding revealed that the NICT group can improve the capability for the immune cell infiltration of the ESCA tissues compared with the NCT group. Next, we used the HPA database to identify the types of cells expressing cytotoxic effector factors (e.g., PRF1, GZMA, and GZMM). As shown in **Figure 3G**, these factors were specially expressed in infiltrating CD8+ T and activated NK cells.

Furthermore, the expression of cytokine IFN-γ and its transcription factors EOMES and TBX21 showed an increased state in the NICT group (**Figure 3D**, *p* < 0.05), indicating the creation of the IFN-γ molecule regulating pathway, as well as the activation of T-cell subtype (i.e., Tc1) and NK cells. Given the findings above, we analyzed the IFN-γ response pathway by GSEA. The results showed that this pathway could be activated in the NICT group while remaining suppressed in the NCT group (**Figure 3E**). Furthermore, the correlation analysis further validated that CD8A and CD8B were correlated with transcription factors of IFN-γ (i.e., EOMES) and the regulatory factor of T cells (i.e., TCIRG1), which indicated that CD8+ T cells were strongly correlated with these effectors and the expression of IFN-γ (**Supplementary Figure S3E**).

The findings above suggested that NICT can significantly enhance the infiltration and activation of immune cells centered with CD8+ T and NK cells in ESCA tissues.

### NICT boosted anti-tumor immunity of Tc1 cells

**Figure 4:**
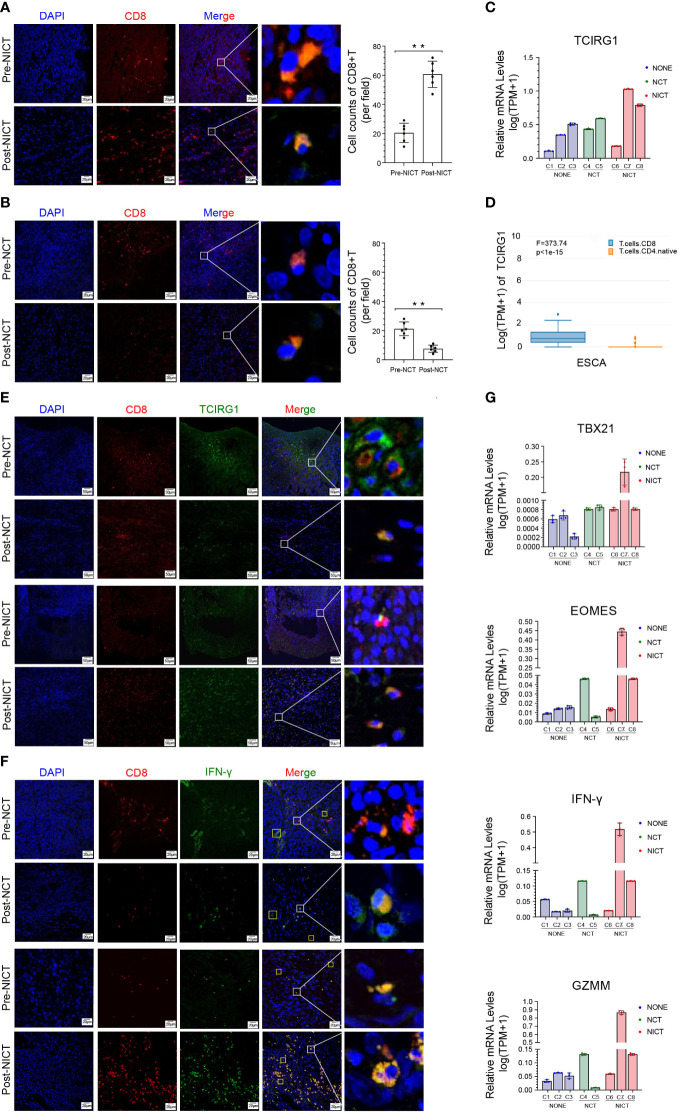
Anti-tumor immunity of CD8+ T cells was boosted by NICT. **(A)** Immunofluorescence showing the expression status and quantity changes in CD8+ T cells in the NICT group before and after PD-1 inhibitor treatment and cell count analysis. **(B)** Immunofluorescence showing the expression status and quantity changes in CD8+ T cells in the NCT group before and after chemotherapy and cell count analysis. **(C)** qRT-PCR showing the expression level of TCIRG1 in each group of clinical cases. **(D)** HAP database showing the types of T cells expressing TCIRG1 in ESCA. **(E)** Multiplex immunofluorescence showing the expression status and quantity changes in CD8+ T cells and TCIRG1 among the NICT and NCT groups pre- and post-NT. **(F)** Multiplex immunofluorescence showing the expression status and quantity changes in CD8+ T cells and IFN-γ among the NICT and NCT groups pre- and post-NT. **(G)** qRT-PCR showing the expression levels of TBX21, EOMES, IFN-γ, and GZMM in clinical cases among the NONE, NCT, and NICT groups. **p < 0.01. NICT, neoadjuvant immunochemotherapy; qRT-PCR, quantitative real-time PCR; ESCA, esophageal cancer; NCT, neoadjuvant chemotherapy; NT, neoadjuvant therapy; NONE, non-neoadjuvant therapy.

PD-1 inhibitors are the T cell-based immune checkpoint blockade, which can recognize the PDCD1 (PD-1, CD279) of CD8+ T cells. The immune response of CD8+ T cells is activated when PDCD1 is blocked (29, 30). First, the expression level of PDCD1 was analyzed in immune cells and esophageal tissues. The results showed that the expression level of PDCD1 was actually higher in some types of CD4 and CD8+ T cells (HPA database, **Supplementary Figures S4A, S4B**), as well as ESCA, but lower in normal esophageal tissues (GEPIA database, **Supplementary Figure S4C**). Therefore, in order to investigate the evidence that NICT increased the clinical remission rate and relied on enhancing the microenvironment of ESCA, we traced the quantity and activation status of CD8+ T cells in the NICT group compared with the NCT group. In the NICT group, we found that the expression and quantity of CD8+ T cells were increased significantly post-NT compared with pre-NT (**Figure 4A**), while they decreased in the NCT group using immunofluorescence technique (**Figure 4B**, *p* < 0.01). The immunohistochemistry (IHC) results also showed that the quantity of CD8+ T cells in the NICT group was significantly higher than that in the NCT group (**Supplementary Figure S4D**, *p* < 0.001).

To explore the regulatory mechanisms of the changes in CD8+ T cells, we further investigated the expression of TCIRG1 as a regulatory factor of T cells. The expression of TCIRG1 exhibited a low level in ESCA and no difference between normal esophageal tissues and ESCA in the GEPIA database (**Supplementary Figure S4E**). In the NICT group, the IHC results suggested that this protein had a certain-level expression in stromal cells (**Supplementary Figure S4F**). Using qRT-PCR for analysis, the expression of TCIRG1 showed clear differences in the pCR and partial response (PR) cases of the NICT group, and it was also related to changes in the tumor size of the NONE and NCT groups (**Figure 4C**). We also identified the expression of TCIRG1 in CD4+ and CD8+ T cells by applying the HAP database and found that it was specifically expressed in infiltrating CD8+ T cells (**Figure 4D**). Moreover, the immunofluorescent staining results suggested that in the NICT group, the expression of TCIRT1 occurred within CD8+ T cells, and the quantity of TCIRG1 was greater post- than pre-NT but decreased in the NCT group (**Figure 4E**).

Tc1 is a subtype of CT8+ T with tumor-killing function, regulated by transcription factors EOMES and TBX21. Because the expression level of IFN-γ can serve as a marker of Tc1 activation, we first analyzed the expression level of IFN-γ of the CD8+ T cells in the ESCA tumor tissues from the NICT and NCT groups. The HPA database showed that the expression was almost nonexistent in ESCA tissues but relatively high in naive CD4+, CD8+ T cells, and activated NK cells (**Supplementary Figure S4G**). Then, we compared the expression of TBX21, EOMES, IFN-γ, and GZMM in the ESCA tumor tissue among the NICT, NCT, and NONE groups by immunofluorescent staining and qRT-PCR. IFN-γ significantly boosted the expression in CD8+ T-cell infiltration in the NICT group (**Figure 4F**). The high-level expression of these four factors was correlated with better tumor remission of patients by qRT-PCR analysis (**Figure 4G**).

The above findings suggested that NICT enabled the activation of the Tc1 in ESCA tissues and expressed a high quantity of IFN-γ and cytotoxic effector factor (GZMM) through the regulation of transcription factors (EOMES and TBX21). Therefore, it constructed the regulatory and functional pathway of CD8+ T activation through the immune infiltration by NICT.

### NICT promoted CD16+ NK cells killing tumor cells

**Figure 5:**
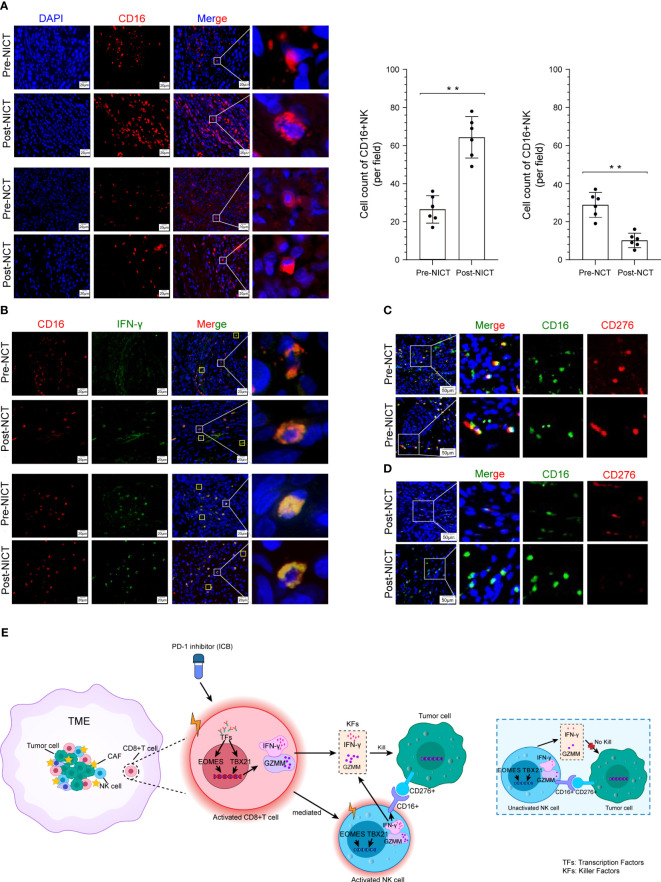
CD16+ NK cells killing tumor cells were promoted depending on NICT. **(A)** Immunofluorescence showing the expression status and quantity changes in CD16+ NK cells among the NICT and NCT groups pre- and post-NT and cell count analysis. **(B)** Multiplex immunofluorescence showing the expression status and quantity changes in CD16+ NK cells and IFN-γ among the NICT and NCT groups pre- and post-NT. **(C)** Multiplex immunofluorescence showing the expression status and quantity changes in CD16+ NK and CD276+ cells among the NICT and NCT groups pre-NT. **(D)** Multiplex immunofluorescence showing the expression status and quantity changes in CD16+ NK and CD276+ cells among the NICT and NCT groups post-NT. **(E)** Pattern diagram of NICT activated the immune cells to kill ESCA cells. **p < 0.01. NICT, neoadjuvant immunochemotherapy; NCT, neoadjuvant chemotherapy; NT, neoadjuvant therapy; ESCA, esophageal cancer.

Except for CD8+ T cells, activated NK cells are another important subtype of cytotoxic cells that play a crucial role in killing esophageal cancer cells. We utilized RNA-seq data (heatmap) to analyze the expression of NK cells’ characteristic antigens and found that these NK cells’ antigens showed different states among the NONE, NCT, and NICT groups (**Supplementary Figure S5A**). Further, we detected the infiltration status of the NK cells in the ESCA tissues by IHC using CD16 as a marker. The IHC results indicated widespread infiltration of CD16+ cells, with greater quantities of CD16+ cells observed in the remaining tumor tissues from the NICT group compared with the NCT group (**Supplementary Figure S5B**). Additionally, comparing the infiltration of CD16+ cells in tumor tissue before and after treatment, it was found that it significantly increased after the use of PD-1 inhibitors in the NICT group (**Figure 5A**, top), while it decreased in the NCT group (**Figure 5A**, bottom). At the same time, we analyzed the expression of IFN-γ in ESCA tissues and found that it significantly increased in the NICT group, but not in the NCT group. IFN-γ is also specially expressed in activated CD16+ NK cells in addition to being expressed in activated CD8+ T cells (**Supplementary Figure S5C**). The immunofluorescence analysis results showed that the detected IFN-γ amounts were mostly in CD16+ NK cells in the NICT group (**Figure 5B**, bottom).

ICBs often exert inhibitory effects on specific markers of immune cells or tumor cells and then activate the immune cells of the TME. However, it remains unclear whether the cytotoxicity of activated immune cells has selectivity in different ICBs. Therefore, applying RNA-seq data, we analyzed the expression of the ICB marker that has been utilized in clinical cancer treatment. We found that the expression of PD-1 (PDCD1/CD279) was lower in ESCA samples and that the differences in the corresponding ligand (tumor cell) CD274 (PD-L1) were non-significant across patients and groups (**Supplementary Figure S5D**). However, B7-H3 (i.e., CD276, a homolog of PD-L1), which is another member of the B7 immunoglobulin superfamily, drew our attention. As shown in **Supplementary Figure S5D**, this molecule exhibited the highest expression among ICB-related molecules in the expression profile data of each group of samples. Searching through the HPA database, we found that CD276 is highly expressed in many tumors and rarely expressed in immune cells (**Supplementary Figures S5E, S5F**). The data from GEPIA and UALCAN demonstrated a high-level expression of CD276 in ESCA tissues (**Supplementary Figures S5G, S5H**). We then utilized CD276 as a marker to detect the infiltration status of the ESCA tissues by IHC, and the results indicated that CD276+ cells also widely infiltrated the ESCA tissues (**Supplementary Figure S5I**). In order to explore the relationship between CD276+ cells and cytotoxicity cells, we further examined the pathology slides of ESCA tissues using CD16, CD8, and CD276 antibodies. Notably, CD276+ cells were found in eight out of nine (88.89%) ESCA lesions in the random testing of each group using immunofluorescence staining, and pre-NT, the results of immunofluorescence co-staining showed that CD16+ NK cells could recognize CD276+ tumor cells in the NCT and NICT groups (red fluorescence and green fluorescence exhibit close-range optical interference phenomenon), as shown in **Figure 5C**. However, post-NT, CD276+ tumor cells still appeared in the NCT group but were reduced significantly in the post-NICT group, while CD16+ NK cells could be observed in the remaining tumors of the NCT and NICT groups (**Figure 5D**). However, there were no such characteristics found with CD8+ T cells (**Supplementary Figures S5J, S5K**). It indicated that CD16+ NK cells that infiltrated tumor lesions could recognize CD276+ tumor cells but with no capability of cytotoxicity before the treatment of PD-1 inhibitors, while it can kill CD276+ tumor cells after the treatment of PD-1 inhibitors.

In summary, these findings showed that CD8+ T cells were activated by PD-1 inhibitors and secreted more IFN-γ to kill ESCA cells. At the same time, activated CD8+ T cells mediated the CD16+ NK cell activation and secreted more IFN-γ to execute the killing function, whereas CD16+ NK cells could not achieve the cytotoxicity of CD276+ tumor cells when the CD8+ T cells were inactive (**Figure 5E**).

## Discussion

In recent years, NT has been widely adopted due to its capability in advancing the effectiveness of clinical cancer treatment, as well as improving the prognosis of cancer patients (29, 31, 32). However, there is still controversy over the options and models of NT for ESCA. Especially in the era of immunotherapy, many solid tumors have shown significant benefits in treatment with the participation of ICBs (30, 33). Similarly, traditional NT for ESCA is also facing challenges from immunotherapy.

ICBs resist tumor cells by engaging the immune system, utilizing the high specificity, monitoring capability, and long-term memory capability to achieve effective and sustained treatment effects. The combined treatment of NT and ICBs (i.e., NICT) is an important strategy in cancer treatment, leveraging the power of the immune system to enhance tumor control, increase the rate of surgical resection, and improve patient prognosis. Whether NICT can be used for the treatment of ESCA is currently one of the forefront research hotspots in clinical practice (34, 35). A study has found that NICT had promising clinical and safety outcomes for patients with resectable ESCA (36). Established evidence showed that patients with ESCA could gain survival benefits from NICT, especially for increasing the successful rates of surgery for locally advanced staged patients (37).

Our clinical data results, obtained by CT, esophagography, PET/CT, and other methods, also showed that ESCA patients exhibited a more effective response rate and clinical stage reduction when treated with NICT compared with NCT. The pCR rate and 2-year survival rate were 22.1% and 70.9%, respectively, in the NICT group, while they were 1.6% and 51.5% in the NCT group, respectively, consistent with recent research results (38–40), suggesting that NICT achieved remarkable benefits in patients with ESCA than NCT. Therefore, for the treatment of ESCA, NICT will have better efficacy and survival benefits and broader future clinical applications.

The efficacy discrepancy between NCT and NICT in ESCA is proposed to be associated with the ICBs, which can restore the monitoring capability of T cells on cancer cells in patients, thus effectively eliminating cancer cells (41). Therefore, we analyzed the clinical remission cases to compare the treatment response between NICT and NCT and discussed the changes in T cells in ESCA lesions. Our findings suggested that there was a significant difference in CD8+ T cells between NCT and NICT in ESCA samples. Post-NCT, the infiltration of CD8+ T cells in the TME significantly decreased, while post-NICT, it significantly increased, indicating that NICT can effectively enhance the anti-tumor immune response by increasing the number of CD8+ T cells, thereby killing tumor cells. It has been reported that the infiltration of CD8+ T within the TME is a key indicator to reflect the effectiveness of immune therapy (42). As the classical cytotoxicity CD8+ T cells, the Tc1 subtype is a unique cytotoxicity cell, which can effectively kill tumor cells (43). Functionally, the Tc1 cell is capable of producing high-level GZMM, IFN-γ, and TNF-α, and their activity is co-regulated by transcription factors TBX21 and EOMES coordinately (44). Classical IFN-γ+ Tc1 cells are the most common subtype in the TME and have been captured in tumor infiltration lymphocytes (TILs), including melanoma, ovarian cancer, breast cancer, and lung cancer. Originally from IFN-γ, prior evidence suggested that the potential of cytotoxicity could be the reason why Tc1 cells are related to better prognoses. Meanwhile, cytokine IFN-γ exerts a direct effect on tumor cells, enhancing its sensitivity to cytotoxicity dependent on CD8+ T cells (45). In fact, the direct effect of IFN-γ on tumor cells is highly related to the anti-tumor effect. Our results indicated that the expression of the IFN-γ and its transcription factors EOMES and TBX21 were increased post-NICT, suggesting that NICT could activate CD8+ T cells and enhance its cytotoxic effect in ESCA. In summary, CD8+ T cells were activated and produced stronger anti-tumor responses post-NICT, reflecting that NICT for ESCA may induce antigen exposure, thereby restoring the anti-tumor immune efficacy.

NK cells are another type of tumor-killing cells that can mediate tumor cell destruction through multiple mechanisms, including releasing perforin/granzyme pathways, secreting IFN-γ and TNF-α, and releasing antibody-dependent cell-mediated cytotoxicity (ADCC) pathways. The activated NK cells express TBX21 and EOMES, further regulating cell cytokines such as IFN-γ and TNF-α, which are the main source of cytokines (e.g., IFN-γ, TNF-α, and GM-CSF) and chemokines. The tumor-killing function could be driven by the release of GZMM to destroy the membrane of tumor cells to trigger apoptosis (46). The activated NK cells can effectively recognize the tumor cells’ surface antigens by expressing the receptors of CD16 (FCγRIIIA), NKp30, and NKG2D. They achieve an anti-tumor effect by the pathway of ADCC and also play a role in ADCC and immune regulation. Results have suggested that NK cells induce an ADCC response in combination with anti-PD-L1 antibody, which promotes ADCC anti-tumor activity against PD-L1-positive tumors (47). Our data demonstrated a significant increase in the proportion of CD16+ NK cells following NICT, suggesting the potential for NK cells to be reactivated by CD8+ T cells and to exert anti-tumor effects after applying PD-1 inhibitors.

In addition to the activation of NK cells, we specifically focused on the antigen recognition and cell killing of NK cells to CD276+ tumor cells. Our results showed that NK cells could recognize CD276+ tumor cells through CD16+, but their killing function was limited in NCT. However, post-NICT, CD16+ NK cells were activated by the activated CD8+ T cells and secreted increased tumor killer factor IFN-γ, thereby effectively killing tumor cells. These results suggested that NICT played an indispensable role in the NT efficacy in ESCA by activating CD8+ T cells and mediating the activation of CD16+ NK cells, enhancing their tumor-killing capabilities.

NICT can sensitize the TIME and play a significant role in promoting therapeutic efficacy by affecting the TME. However, there are still some limitations that need to be addressed. First, the sample size of the mechanism study was limited, which needs further validation. Second, there were notable changes in chemokines among ESCA patients after NICT compared to NCT; for instance, the expression of factors such as IL17A that were secreted by CD4+ T cells also showed dynamic changes before and after different treatments. This suggests that CD4+ T cells also play a role in the treatment process; however, their specific functions are still unknown (48).

In conclusion, NICT has been used in the treatment of ESCA, yet the mechanisms of the action are not clear. In this research, which included 279 clinical cases, we found that individuals in the NICT group showed significant clinical efficacy compared with the NCT group. Mechanism research revealed that NICT could promote the expression of immune-related genes and activate the immune microenvironment. The CD8+ T cells were activated post-NICT, then stimulated CD16+ NK cell activation, and achieved tumor cell killing by cytokines (e.g., IFN-γ and GZMM). In addition, we explored whether CD276 (B7-H3) may be regarded as a new ESCA cell marker that could be recognized by CD16+ NK cells. This study holds significant value in the development of NICT strategies for ESCA.

## Data availability statement

The original contributions presented in the study are publicly available. This data can be found here: Gene Expression Omnibus (GEO), accession number PRJNA1114802.

## Ethics statement

The studies involving humans were approved by the Ethics Committee of Harbin Medical University Cancer Hospital. The studies were conducted in accordance with the local legislation and institutional requirements. The participants provided their written informed consent to participate in this study. Written informed consent was obtained from the individual(s) for the publication of any potentially identifiable images or data included in this article.

## Author contributions

YH: Conceptualization, Data curation, Formal analysis, Investigation, Methodology, Software, Writing – original draft, Writing – review & editing. DY: Data curation, Formal analysis, Methodology, Software, Validation, Writing – review & editing. XL: Conceptualization, Data curation, Formal analysis, Methodology, Software, Writing – original draft. JFZ: Conceptualization, Data curation, Investigation, Resources, Supervision, Validation, Writing – original draft. RC: Conceptualization, Data curation, Formal analysis, Methodology, Software, Validation, Writing – original draft. LC: Data curation, Software, Writing – original draft. LY: Data curation, Writing – original draft. MZ: Data curation, Writing – original draft. XS: Data curation, Writing – original draft. XJ: Data curation, Writing – original draft. HDS: Formal analysis, Writing – original draft. HXS: Formal analysis, Writing – original draft. JYZ: Data curation, Writing – original draft. YL: Data curation, Formal analysis, Methodology, Supervision, Writing – original draft, Writing – review & editing. JM: Data curation, Methodology, Resources, Supervision, Writing – original draft. HN: Data curation, Formal analysis, Funding acquisition, Methodology, Project administration, Supervision, Writing – original draft, Writing – review & editing.
