# Supplementary figures and images for: Neoadjuvant immunochemotherapy improves clinical outcomes of patients with esophageal cancer by mediating anti-tumor immunity of CD8+ T (Tc1) and CD16+ NK cells
Source: Front Immunol. 2024 Jul 15;15:1412693. doi: 10.3389/fimmu.2024.1412693 (PMC11284045; doi:10.3389/fimmu.2024.1412693)

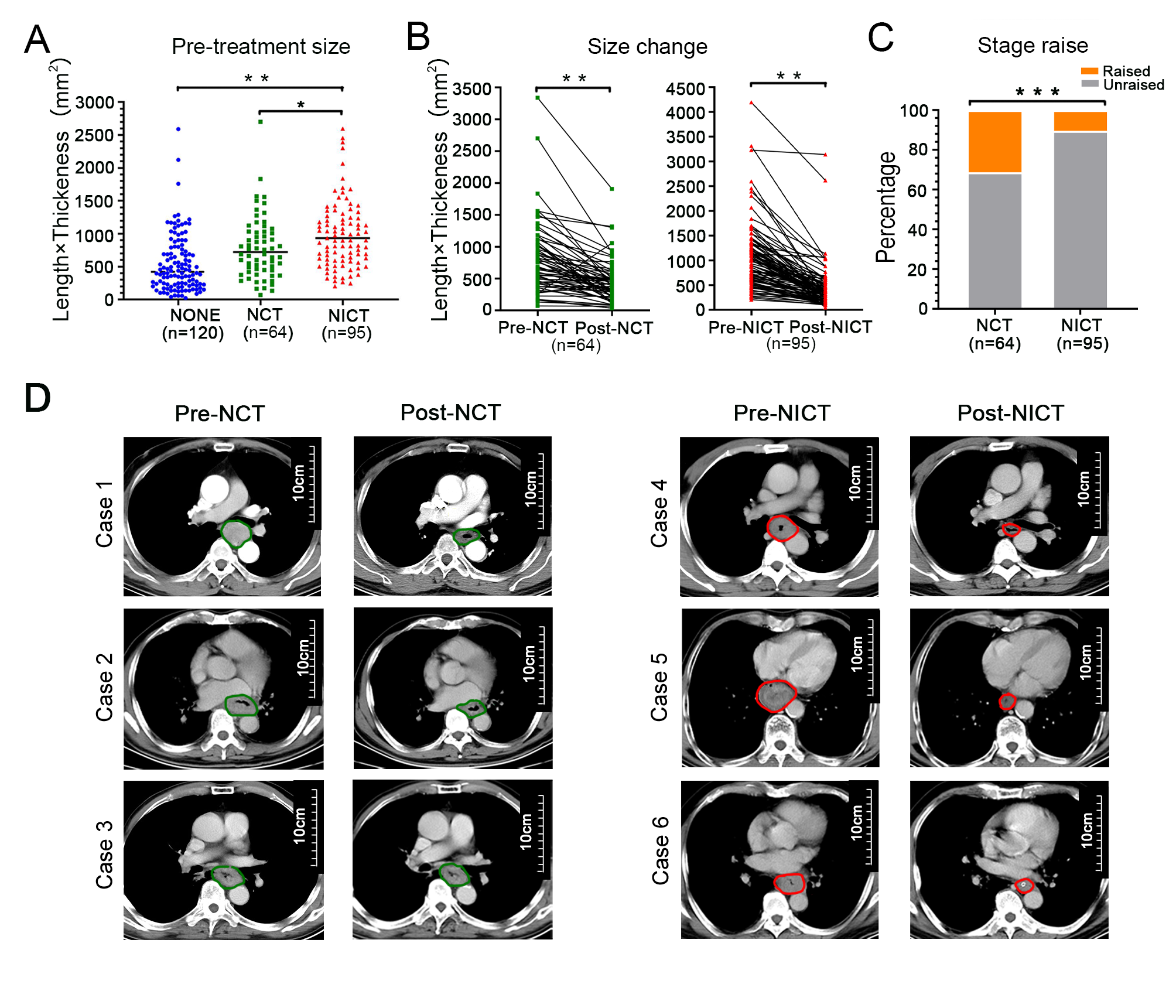

Supplement: Supplementary file 1 [file DataSheet_1.zip › SUPPLEMENTARY FIGURE S1.tif]

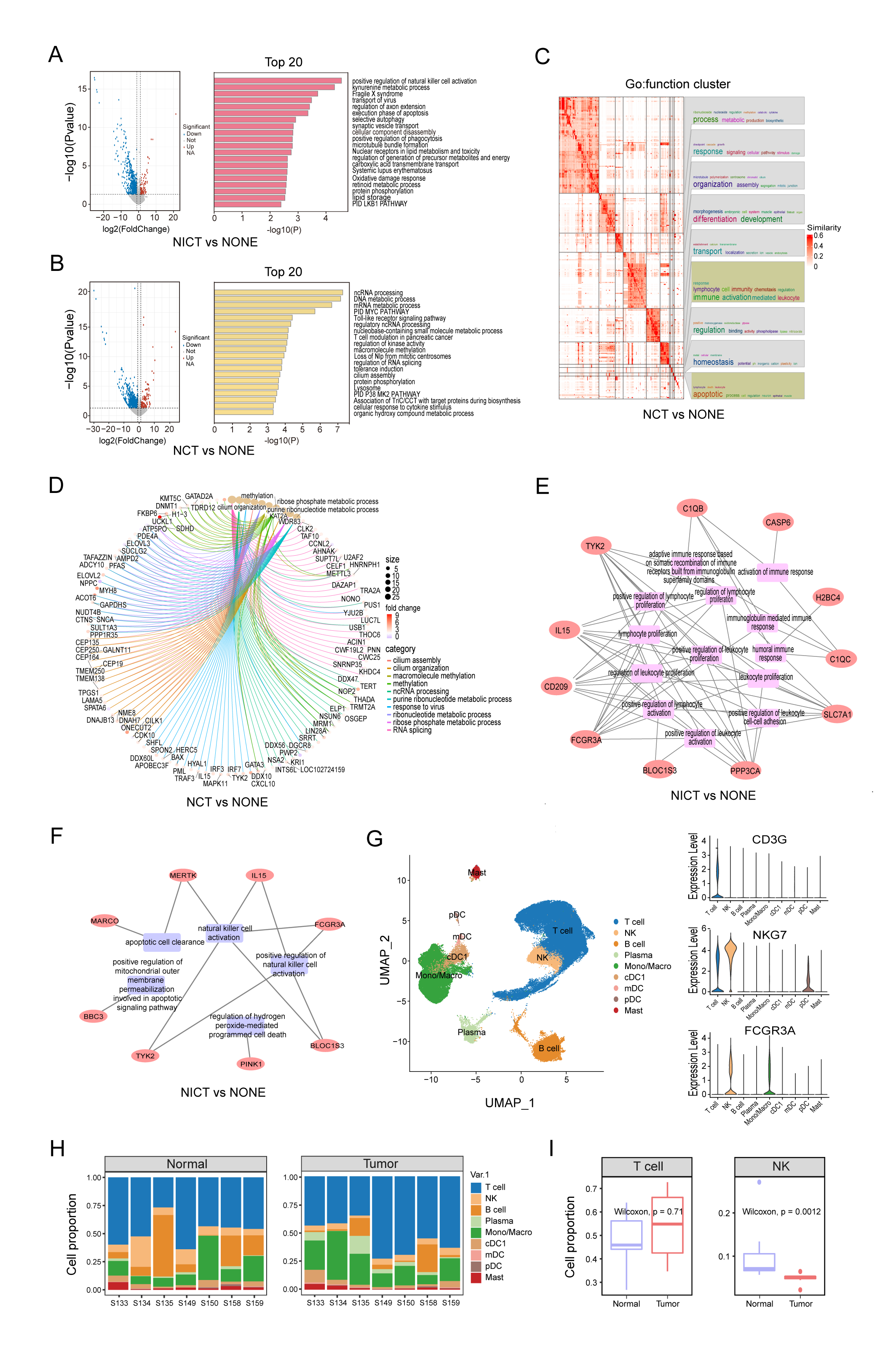

Supplement: Supplementary file 1 [file DataSheet_1.zip › SUPPLEMENTARY FIGURE S2.tif]

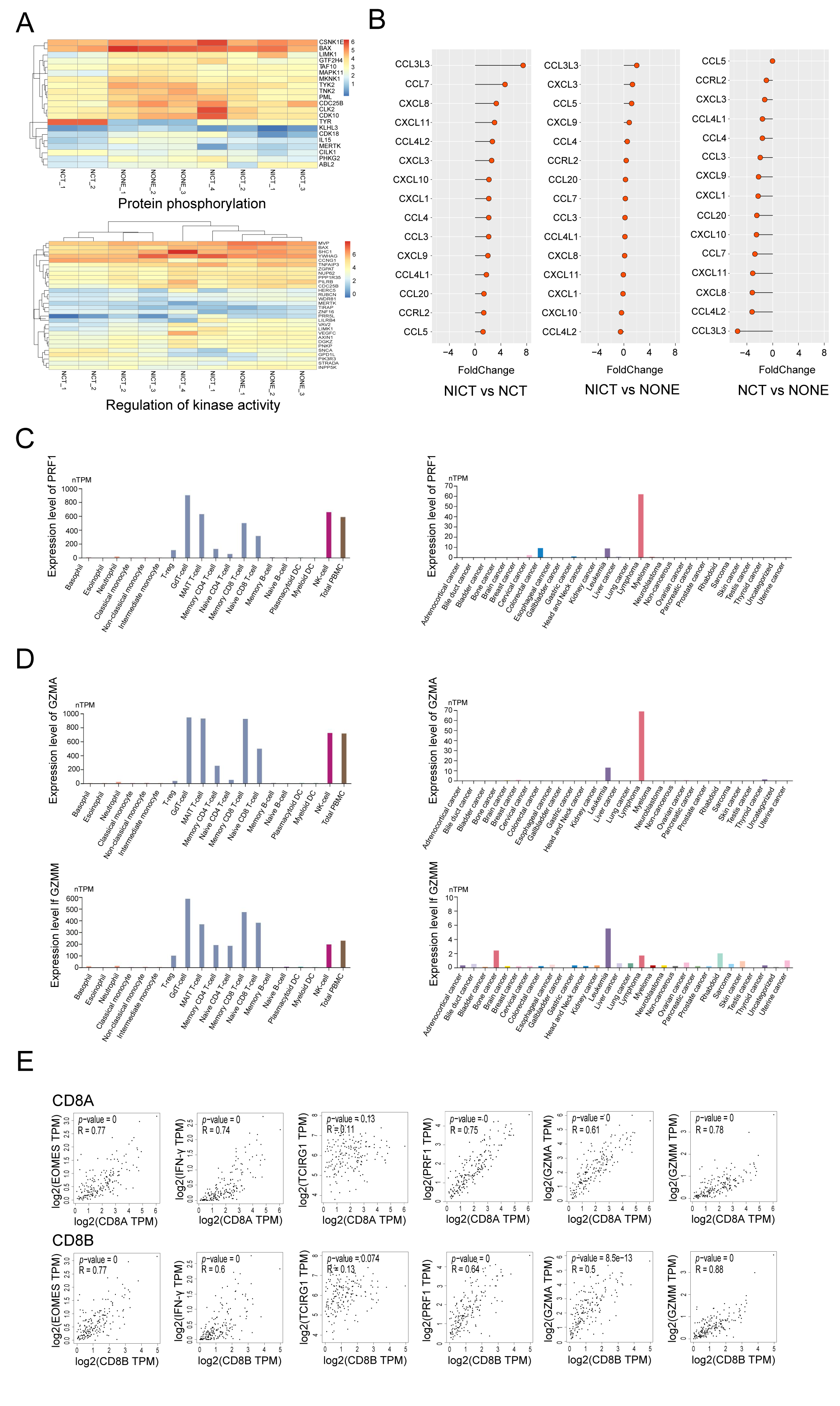

Supplement: Supplementary file 1 [file DataSheet_1.zip › SUPPLEMENTARY FIGURE S3.tif]

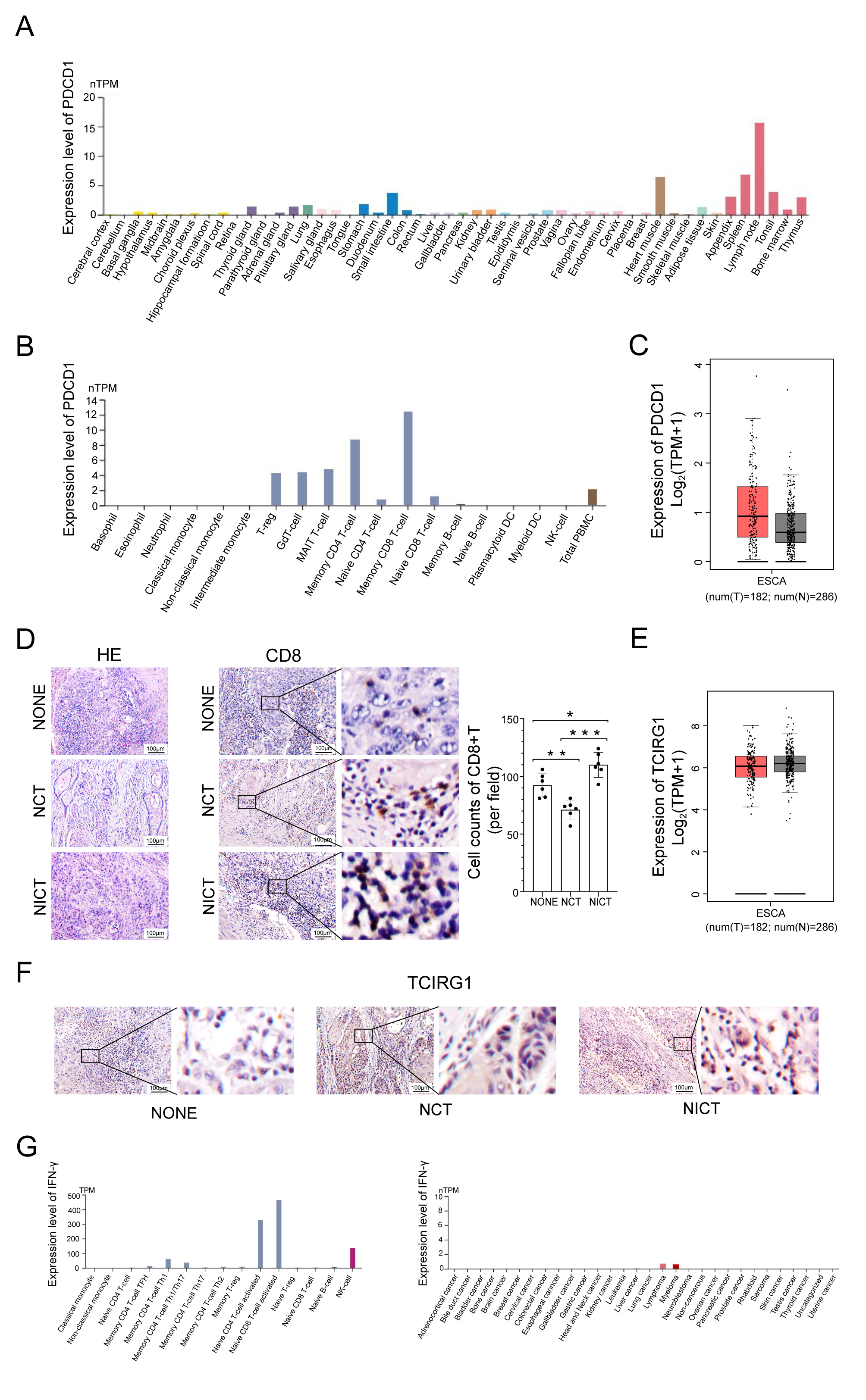

Supplement: Supplementary file 1 [file DataSheet_1.zip › SUPPLEMENTARY FIGURE S4.tif]

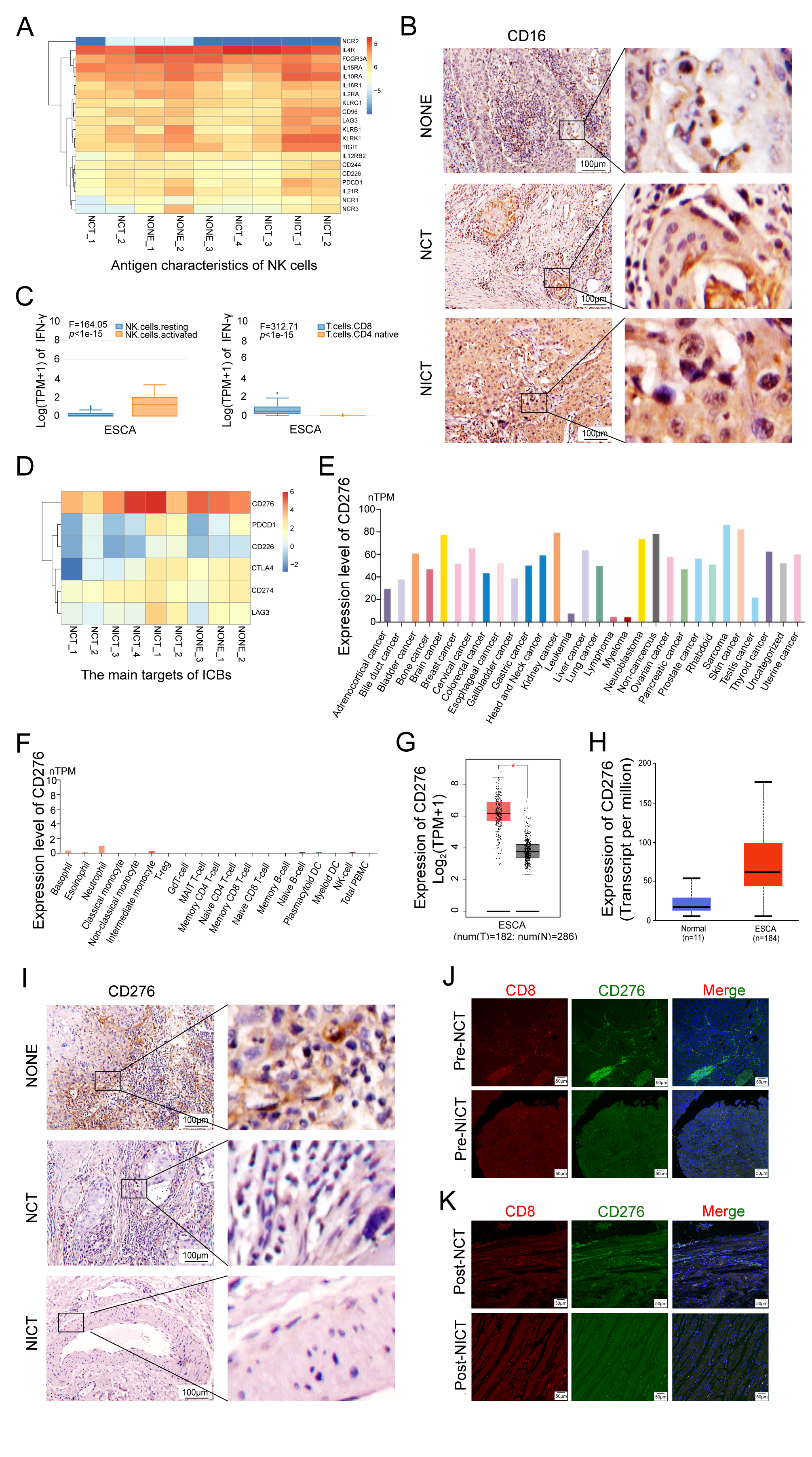

Supplement: Supplementary file 1 [file DataSheet_1.zip › SUPPLEMENTARY FIGURE S5.tif]
