# Supplementary material for: Neoadjuvant immunochemotherapy improves clinical outcomes of patients with esophageal cancer by mediating anti-tumor immunity of CD8+ T (Tc1) and CD16+ NK cells
Source: Front Immunol. 2024 Jul 15;15:1412693. doi: 10.3389/fimmu.2024.1412693 (PMC11284045; doi:10.3389/fimmu.2024.1412693)
Supplement: Supplementary file 2 [file Table_1.docx]

Supplementary Material

# Supplementary Figures and Tables

## Supplementary Figures

SUPPLEMENTARY FIGURE 1

(A) The size of the tumor pre-treatment in each group. NONE, surgery alone; (B) Changes in size pre and post-treatment in the NCT group and NICT group; (C) The ratio of clinical staging raise between NCT group and NICT group; (D) Clinical cases. The maximum thickness of tumors in cross-sectional CT images. **p* < 0.05, ***p* <0.01, ****p* < 0.001.

SUPPLEMENTARY FIGURE 2

(A) Comparison of pathway enrichment analysis between the NONE and NICT groups; (B) Comparison of pathway enrichment analysis between the NONE and NCT groups; (C) Comparison of function cluster of the differential genes between the NONE and NCT groups; (D) Comparison of cycle net plot between the NONE and NCT groups; (E) The network of regulating immune function suggested that the increasing expressions of genes in the NICT group promoted adaptive immune response; (F) The network of regulating immune function shows the increasing expressions of genes in the NICT group can activate and regulate NK cells; (G) Expression of CD3G,NKG7, and FCGR3A in T cells and NK cells analyze by single-cell analysis; (H) The proportion of various types of immune cells in esophageal cancer and normal tissue; (I) The proportion of T cells and NK cells in tumor and normal tissue.

SUPPLEMENTARY FIGURE 3

(A) Heatmap shows the production of protein phosphorylation and regulation of kinase activity in clinical cases among the NONE, NCT, and NICT groups; (B) Compare the expression of cytokines in the NONE group, NCT group, and NICT group; (C) Expres-sion levels of PRF1 in immune cells and tumors based on the Human Protein Atlas(HPA) database; (D) Expression levels of GZMA and GZMM in immune cells and tumors based on HPA database; (E) The correlation between CD8A and CD8B+T cells and the expression of EOMES,IFN-γ,TCIRG1,PRF1,GZMA and GZMM based on HPA database.

SUPPLEMENTARY FIGURE 4

(A) HPA database showed the expression level of PDCD1 in tumors; (B) HPA database showed the expression level of PDCD1 in immune cells; (C) Expression status of PDCD1 in ESCA from GEPIA database; (D) IHC showed the expression status of CT8+T cells among NONE, NCT and NICT groups,and performed cell count analysis; (E) Expression status of TCIRG1 in ESCA from GEPIA database; (F) IHC showed the expression status of TCIRG1 among NONE, NCT and NICT groups; (G) HPA database showed the expression level of IFN-γ in immune cells and tumors. **p* < 0.05, ***p* <0.01, ****p* < 0.001.

SUPPLEMENTARY FIGURE 5

(A) Heatmap shows the expression status of NK cell characteristic antigen in each group of cases; (B) IHC showed the expression status of CD16+NK cells among NONE, NCT and NICT groups; (C) HAP database showed the types of T cells and NK cells expressing IFN-γ in ESCA; (D) Heatmap showed the main targets of ICBs in each group of clinical cases; (E) HAP database showed the expression level of CD276 in many tumors; (F) HAP database showed the expression level of CD276 in immune cells; (G) Expression status of CD276 in ESCA from GEPIA database; (H) Expression status of CD276 in ESCA and the normal tissues from UALCAN database; (I) IHC showed the expression status of CD276 cells in each group; (J) Multiplex immunofluorescence showed the expression status and quantity changes in CD8+ cells and CD276+ cells among the NICT and NCT groups in pre-NT; (K) Multiplex immunofluorescence showed the expression status and quantity changes in CD8+ cells and CD276+ cells among the NICT and NCT groups in post-NT.

1.2 Supplementary Tables

SUPPLEMENTARY TABLE S1 Baseline characteristics of patients in three treatment groups.

| Characteristics | NONE(%)^a^  (n=120) | NCT(%)^b^  (n=64) | NICT(%)^c^  (n=95) |
| --- | --- | --- | --- |
| Sex:  Male  Female | 111 （92.5）  9 (7.5) | 62（96.9）  2（3.1） | 94（98.9）  1（1.1） |
| Age(year):  <60  ≥60 | 46（38.3）  74（61.7） | 27（42.2）  37（57.8） | 49（51.6）  46（48.4） |
| History of smoking  No  Yes | 54（44.8）  66（55.2） | 19（29.2）  45（70.8） | 39（40.8）  56（59.2） |
| History of alcohol  No  Yes | 55（45.9）  65（54.1） | 23（35.4）  41（64.6） | 42（40.8）  61（59.2） |
| Comorbidity^d^  No  Yes | 86（71.3）  34（28.7） | 45（69.2）  19（30.8） | 76（79.6）  19（20.4） |
| Family history  No  Yes | 105（87.5）  15（12.5） | 59（92.2）  5（7.8） | 81（85.3）  14（14.7） |
| ECOG^e^  0  1 | 5（4.2）  115（95.8） | 2（3.1）  62（96.9） | 2（2.1）  93（97.9） |
| Tumor location^f^  Upper  Middle  Lower | 5（4.2）  32（26.7）  83（69.1） | 7（10.9）  24（37.5）  33（51.6） | 6（6.3）  24（25.3）  65（68.4） |
| Pathology  Squamous-cell carcinoma  Adenocarcinoma  Other | 99（86.7）  11（7.7）  10（5.5） | 54（84.6）  5（7.7）  5（7.7） | 91（96.1）  1（1.0）  3（2.9） |
| Differentiation  Well  Moderately  Poorly  Unknown | 9（7.5）  60（50）  44（36.7）  7（5.8） | 5（7.8）  26（40.6）  27（42.2）  6（9.2） | 7（7.4）  36（37.9）  24（25.3）  28（29.4） |
| Clinical T stage  T1  T2  T3  T4a  Could not be determined | 16（13.3）  40（33.3）  63（52.6）  1（0.8）  0（0） | 8（12.5）  11（17.2）  36（56.2）  9（14.1）  0（0） | 2（2.1）  23（24.2）  64（67.4）  6（6.3）  0（0） |
| Clinical N stage  N0  N1  N2  N3  Could not be determined | 51（42.5）  51（42.5）  14（11.7）  0（0）  4（3.3） | 22（34.4）  27（42.1）  14（21.9）  1（1.6）  0（0） | 18（18.9）  52（54.7）  21（22.2）  2（2.1）  2（2.1） |
| Clinical stage^g^  I  II  III  IVA  Could not be determined | 15（12.5）  52（43.3）  48（40.0）  1（0.8）  4（3.3） | 8（12.5）  18（28.1）  26（40.6）  12（18.8）  0（0） | 2（2.1）  28（29.5）  58（61.1）  7（7.4）  0（0） |

a NONE，surgery alone (non-neoadjuvant therapy); b NCT, neoadjuvant chemotherapy; c NICT, neoadjuvant immunochemotherapy; d Comorbidity, including hypertension, cardiovascular and cerebrovascular diseases, diabetes, hepatitis B and C; e ECOG, Eastern Cooperative Oncology Group; f Tumor location, the location of the tumor is mainly determined by gastroscopy,CT and esophagography; g Clinical stage, based on Union for International Cancer Control/American Joint Committee on Cancer 8th edition staging criteria (UICC/AJCC ).

SUPPLEMENTARY TABLE S2 Sequences of qRT-PCR primer

| Primer name | Sequences （5'→3'） |
| --- | --- |
| Q-GAPDH-F | ACAACTTTGGTATCGTGGAAGG |
| Q-GAPDH-R | GCCATCACGCCACAGTTTC |
| Q-TNFAIP3-F | TCCTCAGGCTTTGTATTTGAGC |
| Q-TNFAIP3-R | TGTGTATCGGTGCATGGTTTTA |
| Q-FCGR3A-F | CCTCCTGTCTAGTCGGTTTGG |
| Q-FCGR3A-R | TCGAGCACCCTGTACCATTGA |
| Q-IL17A-F | AGATTACTACAACCGATCCACCT |
| Q-IL17A-R | GGGGACAGAGTTCATGTGGTA |
| Q-IFI30-F | CCCCTCTGCAAGCGTTAGAC |
| Q-IFI30-R | CCCGCAGGTATAGATTGCCT |
| Q-BAX-F | CCCGAGAGGTCTTTTTCCGAG |
| Q-BAX-R | CCAGCCCATGATGGTTCTGAT |
| Q-BACH1-F | TCTGAGTGAGAACTCGGTTTTTG |
| Q-BACH1-R | CGCTGGTCATTAAGGCTGAGTAA |
| Q-CNN2-F | ACCGGCTCCTGTCCAAATATG |
| Q-CNN2-R | CCCGGCTGTAGCTTGTTCA |
| Q-CDK10-F | GCCTGCGTCATCCGAACAT |
| Q-CDK10-R | AGGGTGTTGGCATATTCTCCA |
| Q-TNK2-F | CTGTCCCACTTTGAGTACGTC |
| Q-TNK2-R | GACTTGCGTTTGCACAAGGC |
| Q-TYK2-F | GAACCGGCTGTGTACCGTT |
| Q-TYK2-R | ACGTCATTCACAAACTCATGCTT |
| Q-TYR-F | TGCACAGAGAGACGACTCTTG |
| Q-TYR-R | GAGCTGATGGTATGCTTTGCTAA |
| Q-PPP3CA-F | GCGCATCTTATGAAGGAGGGA |
| Q-PPP3CA-R | TGACTGGCGCATCAATATCCA |
| Q-TCIRG1-F | CATGGTCCTTGCGGAGAACC |
| Q-TCIRG1-R | GCCGGTGTAGATGGAGAACAG |
| Q-TBX21-F | GGTTGCGGAGACATGCTGA |
| Q-TBX21-R | GTAGGCGTAGGCTCCAAGG |
| Q-EOMES-F | GTGCCCACGTCTACCTGTG |
| Q-EOMES-R | CCTGCCCTGTTTCGTAATGAT |
| Q-IFN-γ-F | TCGGTAACTGACTTGAATGTCCA |
| Q-IFN-γ-R | TCGCTTCCCTGTTTTAGCTGC |
| Q-GZMM-F | ACACCCGCATGTGTAACAACA |
| Q-GZMM-R | GGAGGCTTGAAGATGTCAGTG |

SUPPLEMENTARY TABLE S3 Primary antibody and secondary antibody

| Antibody name | Brand | Catalog number |
| --- | --- | --- |
| IFN-γ | ProteinTech | 15365-1-AP |
| TCIRG1 | ProteinTech | 12649-1-AP |
| CD8 | ProteinTech | 66868-1-lg |
| CD16 | ProteinTech | 66779-1-lg |
| CD16 | ProteinTech | 16559-1-AP |
| CD276 | ProteinTech | 14453-1-AP |
| CD276 | ProteinTech | 66481-1-lg |
| Goat anti-Mouse IgG | ProteinTech | SA00001-1 |
| Goat anti-Rabbit IgG | ProteinTech | SA00001-2 |
